# Video-assisted thoracoscopic sympathectomy versus modified Wittmoser method in surgical management of primary hyperhidrosis

**DOI:** 10.1186/s13019-020-01176-1

**Published:** 2020-06-10

**Authors:** Duilio Divisi, Gino Zaccagna, Giovanna Imbriglio, William Di Francescantonio, Andrea De Vico, Mirko Barone, Roberto Crisci

**Affiliations:** Thoracic Surgery Unit, University of L’Aquila, “G. Mazzini” Hospital, Piazza Italia 1, 64100 Teramo, Italy

**Keywords:** Hyperhidrosis, Sympathectomy, Sympathicotomy, Video-assisted thoracic surgery

## Abstract

**Purposes:**

We compared two different surgical methods evaluating the effectiveness of procedures and the quality of life (QoL) of patients.

**Methods:**

From January 2010 to November 2017 we carried out 476 biportal video-assisted thoracoscopic surgery (VATS) approaches of sympathetic chain in 238 patients. One hundred and twenty-nine (54%) patients underwent conventional sympathectomy (CS) while 109 (46%) patients underwent sympathicotomy associated with the division of the rami communicantes (MWT). Quality of Life (QoL) was classified as follows: from 20 to 35 excellent; from 36 to 51 very good; from 52 to 68 good; from 69 to 84 poor; and > 84 very poor.

**Results:**

We noticed statistical significant reduction of complications comparing CS with MWT approaches (chest pain from 36.4 to 4.5%; paresthesias from 8.5 to 3.6%; bradycardia from 28.6 to 10%, respectively). The preoperative and postoperative QoL index evaluation revealed a statistically significant improvement after surgery (CS: 86 ± 2 versus 35 ± 1, *p* = 0.00001; MWT: 85 ± 1 versus 33 ± 2, *p* = 0.00001), with general satisfaction of the two techniques.

**Conclusion:**

Modified Wittmoser method seems to be a valid alternative to conventional sympathectomy, minimizing the percentage rate of complications and showing significant effectiveness in the quality of life improvement.

## Introduction

Primary hyperhidrosis (PHH) involves a series of pathophysiological mechanisms and characterized by an idiopathic, chronic, usually focal, bilateral and symmetrical sweating manifesting itself in all seasons and exacerbated by stress, anxiety, fear or nervousness, as a result of an autonomic dysreflexia [1, 2]. Epidemiologically, PHH affects both sexes without any preference of age of onset, though most cases occur around the third decade of life [3] and a higher prevalence has been reported in Asian populations than in Western ones. However, a complex dysfunction of sympathetic system may be related [4]. Notwithstanding established diagnostic criteria, its real incidence is still poorly clarified due to the reticence of patients and the social impact of the disease that causes underestimation, as supported by increasing prevalence in literature with a recent prevalence of 4.6% in general population [5]. Although PHH is a benign condition, it carries non-negligible effects on quality of life leading to feeling of shame, low self-esteem and psychological distress with subsequent withdrawal from social activities, negatively affecting both social and professional life and only exacerbation leads patient to request care [6, 7]. Conservative strategies for treatment are topical agents, anticholinergic drugs, iontophoresis and the use of botulinum toxin [8], though this latter usually last only three-four months. Moreover, prescription of antiperspirant agents, such as aluminium tetrachloride, has been described in non-responder patients [9]. Among surgical treatments, endoscopic thoracic sympathectomy (ETS) appears an effective approach and first line strategy in palmar forms, with improved quality of life up to 95% [10, 11]. Several techniques have been described with minimally invasive approaches such as single-port ones and promising outcomes both in morbidity and mortality, as required for a benign condition such as PHH. In this regard, Cerfolio et al. [12] did not find any superiority in any of them, being a complete nerve disruption the only aspect to pursue in order to avoid regrowth and symptoms. The aim of this study is to introduce a modification to the Wittmoser’s technique (MWT), evaluating its efficacy and safety by comparing it to conventional sympathectomy (CS). In fact, we considered the Wittmoser technique (WT) which provides the exclusive section of rami communicantes [13] eligible for modification and completing it with the sympathetic trunk section (MWT). The rationale for our decision of modifying the WT was an attempt to verify if a reduction of complications (in particular compensatory hyperhidrosis) and recurrences would be obtained by sectioning both the sympathetic trunk and the rami communicantes.

## Materials and methods

From January 2010 to November 2017, 238 randomized unpaired PHH patients (106 male and 132 female) with a mean age of 24 ± 2 years (range: 16–40 years) were enrolled. Most of them complained of palmar hyperhidrosis (124 patients, 52.10%) or multifocal forms such as palmar-axillary sweating (50 patients, 21.01%). Facial PHH was slightly rare with an actual incidence of 3.78% (9 patients). Diagnosis of primary hyperhidrosis was made according to Hornberger’s criteria [1]. Simultaneously, with the clinical diagnosis, each patient was evaluated about self-perceived disease according to hyperhidrosis disease severity scale (HDSS) questionnaire. A preoperative HDSS score > 3 was mandatory for surgery. All patients underwent preoperative clinical work-up, including blood test evidence, chest X-ray to exclude gross diseases and ECG with cardiological assessment (Table 1) and received informed consent about the above mentioned established surgical technique used according to our Hospital Protocol (00201 Rev.1–2011). The study design was approved by the University of L’Aquila School of Medicine.

**Table 1:** Demographics and surgical indications in 238 PHH patients

Patients and Methods	Characteristics	Number of patients	%
**Gender**	Male	106	44.5
	Female	132	55.5
**Age (mean)**	28 ± 2 years range: 16–40 years	/	/
**PHH Symptoms**	Facial	9	3.78
	Axillary	35	14.71
	Palmar	124	52.10
	Palmar-axillary	50	21.01
	Palmar-plantar	20	8.40
**Technique**	Conventional Sympathectomy	129	54.20
	Modified Wittmoser’s Procedure	109	45.80

### Surgical technique

PHH patients were freely randomized and braced into two cohorts according to surgical technique (129 in the cohort of conventional sympathectomy and 109 in the cohort of modified Wittmoser’s procedure). All procedures were carried out via biportal minimally invasive video-assisted approach (8 mm trocar at the fifth or sixth intercostal space along the posterior axillary line for 0-degree optic and 5 mm or 11.5 mm trocars at the fourth intercostal space for electrocautery or energy devices) under general anaesthesia and selective tracheal intubation. Without carbon dioxide (CO2) insufflation, in both the techniques used, the posterior parietal pleura was opened followed by the dissection and excision of main sympathetic trunk at R3 level for cranio-facial dysreflexia, R3-R4 for palmar and palmar-axillary, R4 for axillary and R3-R5 for palmar and plantar hyperhidrosis. In addition, 109 out of 238 treated patients underwent the resection of rami communicantes and T3 and T4 ganglia based on the level of surgical site. Thus, in 109 patients we used the WT, characterized by selective division of rami communicantes, as previously described [13], associated with the interganglionic trunks energy-given interruption at the level of the chosen ribs (MWT). At the end of the procedure, forced lung re-insufflation with gentle external temporary small-bore catheter aspiration was done without the need of any chest drain placement. All patients were discharged on postoperative day 1.

### Data processing and statistics

Statistical analysis was performed using SPSS version 20.0 software for Windows (IBM, Chicago, USA). Continuous variables were expressed as absolute value, simple percentages, means and standard deviations, whereas categorical ones in terms of frequency and percentage. Statistical differences or correlations were evaluated with χ2 test and unpaired t - test. A *p*-value < 0.05 was considered statistically significant. Technical efficacy and feasibility were evaluated according to Forrest plots with their relative Odds Ratios and 95% Confidential Indexes.

### Quality of life (QoL)

Five levels of satisfaction were considered (excellent, very good, good, poor and very poor), deriving from 20 questions divided into four outcome items according to de Campos criteria [14]. The total score ranged from 20 to 100. QoL was classified as follows: from 20 to 35 excellent; from 36 to 51 very good; from 52 to 68 good; from 69 to 84 poor; and > 84 very poor. The questionnaire was administered 7 days before and 40 days, 6 months and yearly after surgery with an average duration of follow-up of 65 ± 3 months.

## Results

**Fig. 1:**
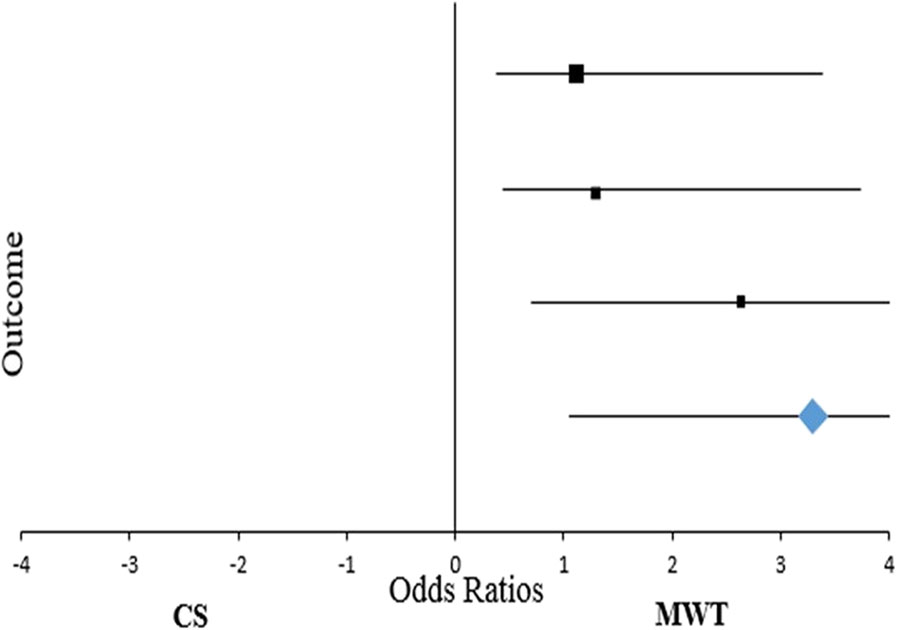
Forrest plot - Conventional Sympathectomy (CS) vs Modified Witmoser’s Procedure (MWT); overall morbidity rates

**Fig. 2:**
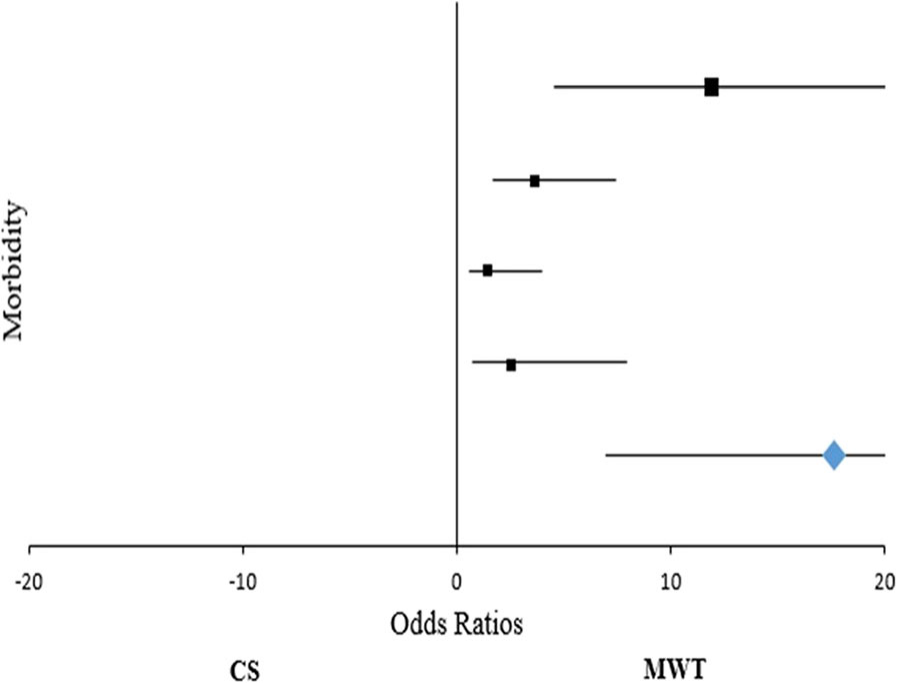
Forrest plot - Conventional Sympathectomy (CS) vs Modified Witmoser’s Procedure (MWT); long-term outcomes

Comparing the two techniques, significant reduction both in operative time (21 ± 1 min vs 13 ± 1 min, *p* <  0.0001) and overall operating room stay (63 ± 2 min vs 42 ± 4 min, *p* <  0.0001) was reported in MWT cohort that, combined with a reduction of both ultrasound (33.33% vs 19.27%, *p* = 0.001) and radiofrequency (35.66% vs 11.92%, *p* < 0.0001) devices, resulted in not negligible cost-effective aspects. However, the reduction of the instrumentation costs does not interfere with patients’ outcome. In particular, MWT is associated with an evident reduction in overall morbidity (24.77% vs 82.94, 95%CI: 46.60–67.20, *p* < 0.0001). Concerning this aspect, the incidence of postoperative thoracic pain, bradycardia and paresthesia evidently decrease due to selective sympathetic denervation rather than classical method (4.59% vs 36.43%, *p* < 0.0001; 10.09% vs 28.68%, *p* = 0.0004; 3.67% vs 8.53%, *p* < 0.0001, respectively; Table 2), as confirmed at Forrest plot analysis with a mean OR of 17.61 (95% CI: 7.02–50.63) favouring the modified approach (Table 3; Fig. 1). However, despite the technique, no statistical differences according to compensatory sweating were found (6.42% vs 9.30%, *p* = 0.415); in fact, this complication disappeared in 45 ± 6 days in our experience. As regards the devices used and their influence on morbidity, energy sources seem to be correlated to an increased percentage of postoperative thoracic pain in CS cohort (25.58% vs 10.85%; *p* = 0.002). Similar results can also be found in the MWT group with evidence tending to significance (3.67% vs 0.92%; *p* = 0.076). Moreover, we noticed two recurrences in CS electrocoagulation patients at the beginning of our experience solved by video-assisted thoracoscopic resurgery in 1 patient, while the other refused reintervention. Electrocautery has been associated to an increased risk of thoracic pain (*p* = 0.001) and bradycardia (*p* = 0.015) in CS procedure when compared with MWT one. Similarly, energy devices seem to predispose to postoperative hyperalgesia (*p* < 0.001) and heart rate abnormalities (*p* = 0.022; Table 4). Finally, concerning patient’s outcome and symptoms’ relief according to de Campos criteria, a statistical improvement of quality of life after surgery with a mean overall satisfactory O.R. of 2.65 (95%CI 0.69–10.05) was reported rather than classical sympathectomy (Table 5; Fig. 2). In fact, patients revealed between poor and very poor Qol before VATS in both CS (86 ± 2) and MWT (85 ± 1) groups. Forty days from intervention Qol was very good (CS Group: 45 ± 2, *p* = 0.00001; MWT Group: 44 ± 1, *p* = 0.00001) and became better at 6th month (CS Group: 35 ± 1, 95% CI between − 51.38 and − 50.61, *p* = 0.00001; MWT Group: 33 ± 2, 95% CI between − 52.42 and − 51.57, *p* = 0.00001). No further changes were recorded during the annual checks. Twenty-four patients were lost at the 4th year of follow up. CS and MWT methods seem to be equivalent being both characterized by sweating relief (93.66% vs 95.00%; *p* = 0.685), improved QoL (93.33% vs 95.00%; *p* = 0.586) and overall satisfaction (93.33% vs 97.33%; *p* = 0.152) (Table 6).

**Table 2:** Conventional Sympathectomy (CS) vs Modified Witmoser’s Procedure (MWT); technical aspects and outcomes

	CS (129 patients)		MWT (109 patients)		95%CI	*p*
	N	%	N	%		
Operative time (minutes)	21 ± 1		13 ± 1		−8.25 - -7.74	< 0.0001
Operating room stay (minutes)	63 ± 2		42 ± 4		−21.78 - -20.21	< 0.0001
Devices						
Electrocautery	40	31.01	75	68.81	25.31–48.60	< 0.0001
Ultrasounds	43	33.33	21	19.27	2.76–24.65	0.001
Radiofrequency	46	35.66	13	11.92	13.35–33.83	< 0.0001
Overall morbidity	107	82.94	27	24.77	46.60–67.20	< 0.0001
Morbidity						
Thoracic pain	47	36.43	5	4.59	22.17–40.81	< 0.0001
Bradycardia	37	28.68	11	10.09	8.564–27.995	=0.0004
Minor compensatory sweating	12	9.30	7	6.42	−4.48 - 9.94	0.415
Paresthesia	11	8.53	4	3.67	−5.93 - 9.54	< 0.0001

**Table 3:** Conventional Sympathectomy (CS) vs Modified Witmoser’s Procedure (MWT); overall morbidity rates

	CS (129 patients)	MWT (109 patients)	O.R.	95%CI
	N	N		
Morbidity				
Thoracic pain	47	5	11.92	4.53–31.33
Bradycardia	37	11	3.58	1.72–7.44
Minor compensatory sweating	12	7	1.49	0.57–3.93
Paresthesia	11	4	2.45	0.76–7.92
Total morbidity			17.61	7.02–50.63

**Table 4:** Postoperative morbidity according to technique and device adopted for surgical method

	CS (129 patients)		MWT (109 patients)		Χ^**2**^			
	Electrocautery	Energy Devices	Electrocautery	Energy Devices	Electrocautery vs Energy Devices (Group A) p	Electrocautery vs Energy Devices (Group B) p	Electrocautery (Group A vs Group B) p	Energy Devices (Group A vs Group B) p
Thoracic pain	14 (10.85%)	33 (25.58%)	1 (0.92%)	4 (3.67%)	0.002	0.076	0.001	< 0.0001
Bradycardia	18 (13.95%)	19 (14.72%)	5 (4.58%)	6 (5.50%)	0.860	0.756	0.015	0.022
Compensatory sweating	3 (2.32%)	9 (6.97%)	2 (1.83%)	5 (4.58%)	0.076	0.250	0.793	0.435
Paresthesia	6 (4.65%)	5 (3.87%)	2 (1.83%)	2 (1.83%)	0.756	1.000	0.229	0.354

**Table 5:** Conventional Sympathectomy (CS) vs Modified Witmoser’s Procedure (MWT); long-term outcomes

	CS (129 patients)	MWT (109 patients)	O.R.	95% CI
	N	N		
Sweating relief	121	103	1.13	0.38–3.38
Improved QoL	120	103	1.28	0.44–3.74
Overall satisfaction	120	106	2.65	0.69–10.05

**Table 6:** Patient’s outcome according to the de Campos questionnaire

	CS (129 patients)	MWT (109 patients)	95% CI	*p*
	N	N		
Sweating relief	121 (93.66%)	103 (95.00%)	−5.2531 to 7.58	0.658
Improved QoL	120 (93.33%)	103 (95.00%)	−4.9710 to 7.98	0.586
Overall satisfaction	120 (93.33%)	106 (97.33%)	−1.9157 to 9.94	0.152

## Discussion

It is the first time a modified Wittmoser’s approach is described. Although selective division of the rami communicantes results in a significant decrease in the rate of disturbing side effects, limited resections usually carry out the high incidence of relapse. In this regard, Gossot et al. [15], in a two-braced retrospective study evaluating classical sympathectomy (54 patients) and Wittmoser’s approach (62 patients), reported a significant risk of recurrence in selective resection group (0 vs 5; *p* < 0.05), though no difference in postoperative compensatory sweating was found (72.2% vs 70.9%). On the other hand, Hwang et al. [16], comparing 46 patients undergoing sympathicotomy and 43 undergoing ramicotomy, found slight significant differences in satisfaction rate (91.3% vs 79.1%, *p* = 0.067) and dryness (82.6% vs 25.6%; *p* < 0.001). Moreover, the incidence of persistent hand sweating and CH was higher in the selective group than that observed in the classical one (16.3% vs 2.2 and 37.2% vs 10.9%). According to this evidence, our proposal was to increase the radicality of ramicotomy by introducing the section of the main trunk without resection of sympathetic ganglia, thus avoiding hypothalamic nervous switch effects at the basis of compensatory dysreflexia phenomena. However, notwithstanding meticulous and accurate dissection, compensatory hyperhidrosis is still the main adverse event after sympathectomy. In our series, postoperative sweating accounts for 7.98% of patients (6.42% for MWT and 9.30% for CS), which is significantly inferior to an overall published incidence ranging from 10 and 78% [17], being an unmodifiable complication as suggested by Shoenfeld et al. [18]. In fact, compensatory sweating should be considered as a para-physiological rearrangement of the organism to the procedure by an increased sweating in other parts of the body without a change in the total amount of sweat, mediated by hypothalamus-induced reflexes leading to the failure of feedback mechanism of the sectioned chains. In particular, a response of the hypothalamus to peripheral stimuli would lead to negative adaptation due to the absence of afferent fibers and to a switch effect to other unsympathectomized areas. Moreover, there seems to be a direct correlation between the severity of CH and the level and the extension of resection, as reported by Cerfolio et al. [12]. In fact, an injury to T2 ganglion with a total interruption of the afferent negative stimulus to hypothalamus favors the emergence of severe CH on the periphery, resulting in refractory dysreflexia whose incidence is about 5% [19]. For these reasons, the variability of the incidence of compensatory hyperhidrosis is due to the different approaches to thoracic ganglia. Ibrahim et al. [20], performing T2-T4 sympathectomy in 130 patients, reported 19% (25 patients) of CH cases. Similar results were published by Turkylmaz et al. [21], in a retrospective study comparing T2 and T3 sympathectomy for palmar hyperhidrosis, with a mean postoperative sweating of 10% (12% T2 group and 8% T3 group). On the other hand, CH incidence is quite higher after reversible techniques as sympathetic clipping. As reported by Joo et al. [22], in 101 patients undergoing thoracoscopic sympathetic surgery, the rate of life-bothering compensatory sweeting was lower in R4 sympathicotomy group compared to R4 or R3 sympathetic clipping ones (0% vs 25% vs 47.8%) with a similar distribution concerning rate of failure (12.50% vs 35.00% vs 34.80%). However, postoperative sweating seems to be also influenced by the planning of treatment. As reported by Menna et al. [23], in a 270 endoscopic thoracic sympathectomy cohort, patients undergoing one-stage bilateral sympathectomy developed CH in 21.4% of cases, while significantly reduced in two-stages (4.4%) with a recovery rate favouring this latter (83.30% vs 33.35%). Regarding operative devices and their effects on patients’ outcome, Kuhajada et al. [24] retrospectively reviewing 70 patients undergoing bilateral thoracoscopic sympathectomy and evaluating their outcome according to the energy device adopted (39 patients with electric scalpel and 40 with harmonic one). Authors did not find any difference in postoperative pain between the two groups (*p* > 0.05), since majority of them claimed that pain lasted one-two weeks after the procedure (69.2% in electric cohort and 50.0% in harmonic one). Intensity of postoperative pain was similar in both groups and mean visual-assessment score pain was four (17.9% electric group and 37.5% harmonic group). However, long-lasting severe pain occurred preferentially in ultrasound generator hook patients (*p* < 0.05). In one of our previous reports on sixty-five patients [25], we found energy devices offered greater efficacy in the treatment of hyperhidrosis and, in particular, latest generations seemed to reduce the incidence of pain by a half, with a significant improvement of quality-adjusted life year. From current results, energy devices seem to be related to an increased incidence of postoperative thoracic pain both in CS cohort (25.58% vs 10.85%; *p* = 0.002) and MWT one (3.67% vs 0.92%; *p* = 0.076). Probably, this aspect can be explained referring to a greater diameter of trocar employed due to the need for a second device able to move the sympathetic chain upwards resulting in a dimension-related encumbrance of the device stem on the thoracoscopic port with an increased risk of intercostal bundle compression and stretching. Another aspect to be investigated is postoperative bradycardia. Both electrocautery and energy devices, in CS, seem to predispose to heart rhythm abnormalities (13.95% vs 4.58%, *p* = 0.015 and 14.72% vs 5.50%, *p* = 0.022, respectively) when compared with MWT technique. However, the reasons are to be found in anatomical aspects rather than device peculiarities. In fact, as reported by Ibrahim et al. [20], ETS significantly interfere with heart rate (77 bpm vs 69 bpm, *p* < 0.001), due to simultaneous cardiac sympathetic denervation [26]. Kim et al. [27] showed a distinctive decrease at rest-heart rate after levels T2, T3, T4 ETSs, as an effect similar to b-blocker treatment inducing a shift of rhythm toward parasympathetic predominance. Moreover, these effects were associated with the extent of denervation being more evident in the sympathectomy group than in the sympathicotomy group, especially after T3 ganglia excision as being this latter the direct pathway of sympathetic innervation of the heart. Thus, the simple disconnection of sympathetic chain between the T2 and T3 ganglia could cause a shift of sympathovagal balance toward vagal activity. So, according to this evidence, our results and the propensity to bradycardia after classical sympathectomy are justified by surgical technique, especially in case of R3 resections. Because of this evidence, our propensity for a two-phase strategy found its rationale in allowing a sympathetic autonomic adaptation to avoid fearsome bradycardias and secondary cardiac arrests at denervation. Concerning postoperative quality of life, results show that both techniques are characterized by satisfying and comparable outcomes without significant differences between the devices used. In particular, overall general satisfaction rate was 93.33% for CS cohort and 97.33% for MWT one which is significantly superior than 88.4% reported by Silva et al. [20] in a retrospective study on 127 PHH patients.

## Conclusions

Although our study requires an external validation on a larger number of patients, the modified Wittmoser’s technique seems to be safer than classical sympathectomy for the treatment of primary hyperhidrosis, as confirmed by an overall reduction in postoperative morbidity. This method consists in the extensive but targeted denervation that, associated with the upper limit of the surgical site not beyond the third rib, eliminates recurrences and reduces complications especially bradycardia, compensatory sweating and Horner’s syndrome. A wider use of the electrocautery in MWT cohort allowed a quality of life substantially equal to the CS group, saving on the cost of the device.

## Data Availability

Yes

## Abbreviations

PHH: Primary Hyperhidrosis
ETS: Endoscopic Thoracic Sympathectomy
CS: Conventional Sympathectomy
MWT: Modified Wittmoser’s technique
CH: Compensatory Hyperhidrosis
